# Supplementary material for: Development of a core outcome set to determine the overall treatment success of acute uncomplicated appendicitis in children: a study protocol
Source: BMJ Paediatr Open. 2017 Aug 11;1(1):e000151. doi: 10.1136/bmjpo-2017-000151 (PMC5862231; doi:10.1136/bmjpo-2017-000151)
Supplement: Supplementary file 1 [file bmjpo-2017-000151supp001.pdf]

APPENDIX 1. Systematic review of published outcomes of conservative treatment of acute appendicitis in children

All papers identified by our systematic review of conservative treatment[1] were included, and the literature search in that paper repeated to identify any further papers published between 2016 and April 2017.

Table 1. Characteristics of included studies

Study	Publication Year	Region	Study Type	Sample size (intention to treat non-operatively)	Age of included population
Abes[2]	2007	Antibiotics	Retrospective	Middle East	16
Armstrong[3]	2014	Antibiotics vs. appendectomy	Retrospective	North America	12
Caruso[4]	2017	Antibiotics vs. appendectomy	Retrospective	Europe	197
Chen[5]	2016	Antibiotic	Retrospective	Asia	125
Gorter[6]	2015	Antibiotics	Prospective	Europe	25
Hartwich[7]	2016	Antibiotics	Prospective	North America	24
Jimbo[8]	2016	Antibiotics	Retrospective	Asia	71
Kaneko[9]	2004	Antibiotic	Prospective	Asia	91
Koike[10]	2014	Antibiotics	Retrospective	Asia	134
Mahida[11]	2016	Antibiotics	Prospective	North America	5
Minecci[12]	2016	Antibiotics	Prospective	North America	37
Mudri[13]	2017	Antibiotics and appendectomy	Retrospective	North America	26
Steiner[14]	2015	Antibiotics	Retrospective	Middle-east	45
Steiner[15]	2017	Antibiotics	Retrospective	Middle-east	197
Svensson[16]	2015	Antibiotics and appendectomy	Prospective RCT	Europe	24
Tanaka[17]	2015	Antibiotics and appendectomy	Retrospective	Japan	78

Outcomes from these papers were then mapped, where possible, to the outcomes identified in our previous systematic review of outcomes of appendicitis in children[18].

Table 2. Outcomes mapped to those previously described[18]

	Abes	Armstrong	Caruso	Chen	Gorter	Hartwich	Jimbo	Kaneko	Koike	Mahida	Minecci	Mudri	Steiner 2015	Steiner 2017	Svensson	Tanaka
**Wound Infection**			x		x								x			
**Intra-abdominal abscess**			x	x	x							x				
**(Adhesive) obstruction**			x													
**Complication of antibiotics or treatment intervention**					x											
**Major/minor complications**		x														
**Non-infectious wound complications**																
**Re-admission to hospital**		x	x		x	X					x	x				
**Other infectious complications**		x		x								x				
**Interventional radiology procedure**																
**Need for operation/re-operation**			x		x	x	x	x		x	x	x	x	x	x	x
**Conversion laparoscopic to open surgery**																
**Recurrent appendicitis**	x	x			x	x	x	x	x	x	x	x	x	x	x	x
**Other complications**			X		x											
**Bacterial isolates**																
**Post-treatment fever**													x	x		
**Measure of recovery of gastro-intestinal function**							x									
**Blood markers (WCC/CRP)**			x										x	x		
**Duration of abdominal drainage**																
**Validated pain score**																
**Other pain assessment**	x		x										x		x	

**Time away from normal activities or school**											x					
**Recovery to full activity/sport**																
**Cosmesis**																
**Time to ambulation**																
**Other PROM**																x
**Duration of home healthcare**																x
**Paediatric quality of life assessment**						x					x					
**Parental quality of life assessment**						x					x					
**Hospital length of stay**		x	x	x			x		x		x	x	x	x	x	x
**Duration of surgery/analgesia**							x									
**Total charges**						x			x		x	x			x	
**Duration of antibiotics**														x		
**Narcotic/analgesia doses**																
**Healthcare visits**		x														
**Post-treatment imaging**	x		x					x					x	x		x
**Cost effectiveness**						x	x					x				
**Death**																

No completely new outcomes were identified using this search. However, perforation is considered as a major complication, and it may be required to elevate this to the level of a separate outcome in the Core outcome set.

References

1. Georgiou R, Eaton S, Stanton MP, et al. Efficacy and Safety of Nonoperative Treatment for Acute Appendicitis: A Meta-analysis. Pediatrics 2017;**139**(3).
2. Abes M, Petik B, Kazil S. Nonoperative treatment of acute appendicitis in children. J Pediatr Surg 2007;**42**(8):1439-42.
3. Armstrong J, Merritt N, Jones S, et al. Non-operative management of early, acute appendicitis in children: Is it safe and effective? Journal of Pediatric Surgery 2014;**http://dx.doi.org/10.1016/j.jpedsurg.2014.02.071**.
4. Caruso AM, Pane A, Garau R, et al. Acute appendicitis in children: not only surgical treatment. J Pediatr Surg 2017;**52**(3):444-48.
5. Chen CL, Chao HC, Kong MS, et al. Risk Factors for Prolonged Hospitalization in Pediatric Appendicitis Patients with Medical Treatment. Pediatrics and neonatology 2017;**58**(3):223-28.
6. Gorter RR, van der Lee JH, Cense HA, et al. Initial antibiotic treatment for acute simple appendicitis in children is safe: Short-term results from a multicenter, prospective cohort study. Surgery 2015;**157**(5):916-23.
7. Hartwich J, Luks FI, Watson-Smith D, et al. Nonoperative treatment of acute appendicitis in children: A feasibility study. J Pediatr Surg 2016;**51**(1):111-6.
8. Jimbo K, Takeda M, Miyata E, et al. Is a pediatrician performed gray scale ultrasonography with power Doppler study safe and effective for triaging acute non-perforated appendicitis for conservative management? J Pediatr Surg 2016;**51**(12):1952-56.
9. Kaneko K, Tsuda M. Ultrasound-based decision making in the treatment of acute appendicitis in children. J Pediatr Surg 2004;**39**(9):1316-20.
10. Koike Y, Uchida K, Matsushita K, et al. Intraluminal appendiceal fluid is a predictive factor for recurrent appendicitis after initial successful non-operative management of uncomplicated appendicitis in pediatric patients. Journal of Pediatric Surgery 2014;**49**(7):1116-21.
11. Mahida JB, Lodwick DL, Nacion KM, et al. High failure rate of nonoperative management of acute appendicitis with an appendicolith in children. J Pediatr Surg 2016;**51**(6):908-11.
12. Minneci PC, Mahida JB, Lodwick DL, et al. Effectiveness of Patient Choice in Nonoperative vs Surgical Management of Pediatric Uncomplicated Acute Appendicitis. JAMA Surg 2016;**151**(5):408-15.
13. Mudri M, Coriolano K, Butter A. Cost analysis of nonoperative management of acute appendicitis in children. J Pediatr Surg 2017.
14. Steiner Z, Buklan G, Stackievicz R, et al. A role for conservative antibiotic treatment in early appendicitis in children. J Pediatr Surg 2015;**50**(9):1566-8.
15. Steiner Z, Buklan G, Stackievicz R, et al. Conservative treatment in uncomplicated acute appendicitis: reassessment of practice safety. Eur J Pediatr 2017;**176**(4):521-27.
16. Svensson JF, Patkova B, Almstrom M, et al. Nonoperative treatment with antibiotics versus surgery for acute nonperforated appendicitis in children: a pilot randomized controlled trial. Ann Surg 2015;**261**(1):67-71.
17. Tanaka Y, Uchida H, Kawashima H, et al. Long-term outcomes of operative versus nonoperative treatment for uncomplicated appendicitis. J Pediatr Surg 2015;**50**(11):1893-7.
18. Hall NJ, Kapadia MZ, Eaton S, et al. Outcome reporting in randomised controlled trials and meta-analyses of appendicitis treatments in children: a systematic review. Trials 2015;**16**:275.
