# Supplementary material for: Development of a core outcome set to determine the overall treatment success of acute uncomplicated appendicitis in children: a study protocol
Source: BMJ Paediatr Open. 2017 Aug 11;1(1):e000151. doi: 10.1136/bmjpo-2017-000151 (PMC5862231; doi:10.1136/bmjpo-2017-000151)
Supplement: Author's manuscript [file bmjpo-2017-000151.draft_revisions.pdf]

BMJ Paediatrics Open

BMJ Paediatrics Open is committed to open peer review. As part of this commitment we make the peer review history of every article we publish publicly available.

When an article is published we post the peer reviewers' comments and the authors' responses online. We also post the versions of the paper that were used during peer review. These are the versions that the peer review comments apply to.

The versions of the paper that follow are the versions that were submitted during the peer review process. They are not the versions of record or the final published versions. They should not be cited or distributed as the published version of this manuscript.

BMJ Paediatrics Open is an open access journal and the full, final, typeset and author-corrected version of record of the manuscript is available on our site with no access controls, subscription charges or pay-per-view fees (http://bmjpaedsopen.bmj.com).

If you have any questions on BMJ Paediatrics Open's open peer review process please email info.bmjpo@bmj.com

BMJ Paediatrics Open

Confidential: For Review Only

Development of a core outcome set for use in determining the overall success of treatment of acute uncomplicated appendicitis in children: study protocol

Journal:	*BMJ Paediatrics Open*
Manuscript ID	bmjpo-2017-000151
Article Type:	Protocol
Date Submitted by the Author:	01-Jun-2017
Complete List of Authors:	Sherratt, Frances; University of Liverpool, Department of Psychological Sciences<br>Eaton, Simon; UCL Institute of Child Health, Developmental Biology and Cancer Programme<br>Walker, Erin; Great Ormond Street Hospital For Children NHS Trust<br>Beasant, Lucy; University of Bristol School of Social and Community Medicine, Centre for Child and Adolescent Health<br>Blazeby, Jane; University of Bristol School of Social and Community Medicine, Centre for Surgical Research<br>Young, Bridget; University of Liverpool, Department of Psychological Sciences<br>Crawley, Esther; University of Bristol School of Social and Community Medicine, Centre for Child and Adolescent Health<br>Wood, Wendy; University of Southampton, NIHR Research Design Services South Central<br>Hall, Nigel; University of Southampton, University Surgery Unit, Faculty of Medicine
Keywords:	Paediatric Practice, Paediatric Surgery, Measurement

SCHOLARONE™
Manuscripts

Development of a core outcome set for use in determining the overall success of treatment of acute uncomplicated appendicitis in children: study protocol

Frances C Sherratt[1], Simon Eaton[2], Erin Walker[3], Lucy Beasant[4], Jane M Blazeby[5], Bridget Young[1], Esther Crawley[4], Wendy W Wood[6], Nigel J Hall[7]

1. Institute of Psychology, University Of Liverpool, Liverpool, UK

2. Developmental Biology and Cancer Programme, UCL Great Ormond Street Institute of Child Health, London, UK

3. Great Ormond Street Hospital NHS Foundation Trust, London, UK

4. Centre for Child and Adolescent Health, School of Social and Community Medicine, University of Bristol, Bristol, UK

5. Centre for Surgical Research, School of Social & Community Medicine, University of Bristol, Bristol, UK

6. Research Design Services South Central, University of Southampton, Southampton, UK

7. University Surgery Unit, Faculty of Medicine, University of Southampton, Southampton, UK

Corresponding author:

Dr Frances C Sherratt

Department of Psychological Sciences, University of Liverpool, Whelan Building, Liverpool, L69 3GB

E-mail: sherratt@liverpool.ac.uk

Tel: +44151 795 0347

Word count, excluding title page, abstract, references, figures and tables: 2,496

Keywords: appendicectomy, appendectomy, appendicitis, antibiotics, children, paediatric, core outcomes, core outcome set, Delphi, consensus meeting

Confidential: For Review Only

ABSTRACT

Introduction

In recent years, there has been growing interest in alternatives to appendicectomy. In particular, non-operative treatment of appendicitis, with antibiotics alone, has been proposed as a potential treatment. A small number of randomised controlled trials (RCTs) in adults and, more recently, children suggest that antibiotic treatment may be a valid alternative to appendicectomy. However, there is currently insufficient data to justify its widespread use. Prior to performing further efficacy studies of the treatment of appendicitis in children, it is imperative to identify the most relevant outcome measures for inclusion in the design of comparative studies. This is of particular importance when evaluating a novel treatment approach since the outcomes of importance may differ from those commonly reported with traditional therapies.

A review of the relevant literature and electronic resources failed to identify a Core Outcome Set (COS) for children with appendicitis. We aim to define a COS for the measurement of treatment interventions in children (< 18 years) with acute appendicitis

Methods and analysis

This project will entail: (i) a systematic review to identify previously reported acute uncomplicated appendicitis treatment outcomes; (ii) assembly of stakeholder panels (paediatric and adult surgeons, patients and parents); (iii) a three stage Delphi process, and; (iv) a final consensus meeting to complete the COS.

Ethics and Registration

COS development is part of CONservative TReatment of Appendicitis in Children - a randomised controlled Trial (Feasibility) - CONTRACT study, for which full ethical approval for CONTRACT has been granted. The COS development study is registered with the COMET initiative in May 2017 (http://www.comet-initiative.org/studies/details/987).

KEY MESSAGES

What is known about the subject?

- Traditionally, appendicectomy has been the gold standard treatment for acute appendicitis in children but there has been increased interest in non-operative treatment (with antibiotics).
- Core outcome sets are developed and adopted to avoid inconsistencies in outcome selection, measurement and reporting that may otherwise exist.
- There is currently no core outcome set for the measurement of effectiveness of treatment interventions in children with acute uncomplicated appendicitis.

What this study adds:

- This project will involve defining a core outcome set for the measurement of effectiveness of treatment interventions in children with acute uncomplicated appendicitis.
- Considering outcomes of importance to patients, parents of patients and health professionals is crucial for paediatric appendicitis research to be meaningful and relevant.

Confidential: For Review Only

BACKGROUND

Acute appendicitis is the most common surgical emergency in children.[1] The lifetime risk of developing appendicitis is 7-8 % with a peak incidence in the early teenage years. Traditionally, appendicectomy has been the gold standard treatment for acute appendicitis. This requires an inpatient stay, a surgical operation requiring general anaesthesia, and exposure to potential complications not only of the disease but also surgery. Whilst the majority of children recover from acute appendicitis, the disease carries a mortality of 0.08-0.31 per 1,000 cases in children.[2]

In recent years, there has been growing interest in alternatives to appendicectomy. In particular, non-operative treatment of appendicitis, with antibiotics alone, has been proposed as a potential treatment. A small number of randomised controlled trials (RCTs) in adults[3-5] and, more recently, RCTs and prospective preference studies in children[6 7] suggest that antibiotic treatment may be a valid alternative to appendicectomy.[8] However, there is currently insufficient data to justify its widespread use. Prior to performing further efficacy studies of the treatment of appendicitis in children, it is imperative to identify the most relevant outcomes for inclusion in the design of comparative studies. This is of particular importance when evaluating a novel treatment approach since the outcomes of importance may differ from those commonly reported with traditional therapies.

Core Outcome Sets (COS) have been proposed as a means of standardizing outcome selection, measurement and reporting in clinical trials.[9 10] The development of a COS and its adoption by researchers is intended to help avoid inconsistencies in outcome selection, measurement and reporting that may otherwise exist. The lack of a COS may result in: (i) important outcomes being overlooked or omitted in study design; (ii) inconsistent definitions or measurement techniques being used across studies, and; (iii) omission of important outcomes from reports (outcome reporting bias).[9] Selecting outcomes that are important to a range of stakeholders is important if research is to be meaningful and relevant. If trials do not adopt an established COS they risk selecting suboptimal outcomes and are unlikely to contribute usable information.[11] A review of the relevant literature and electronic resources failed to identify a COS for children with appendicitis. This project will entail a Delphi process and consensus meeting to define a COS for the measurement of effectiveness of treatment interventions in children (< 18 years) with acute uncomplicated appendicitis.

METHODS

The COS development is a component of a wider project, CONservative TReatment of Appendicitis in Children - a randomised controlled Trial (Feasibility) - CONTRACT study (http://www.nets.nihr.ac.uk/projects/hta/1419290). The COS development study was registered with the COMET initiative in May 2017 (http://www.comet-initiative.org/studies/details/987).

Scope of the COS

The COS is intended to be used to evaluate the overall success of treatment of acute uncomplicated appendicitis, including both operative and non-operative management, including in future research evaluating treatments (of all modalities) of children with acute uncomplicated appendicitis. The COS will include outcome measures identified as important within 12 months following initiation of treatment and longer term outcomes if applicable. The COS focuses specifically on treatment of acute uncomplicated appendicitis; the treatment of perforated appendicitis (with or without abscess) and appendix mass is outside the scope of this COS. The key objectives of the study are:

1. To determine which outcomes have previously been reported in studies comparing treatments for acute uncomplicated appendicitis in children.

https://mc.manuscriptcentral.com/bmjpo

Confidential: For Review Only

2. To prioritise treatment outcomes of children with acute uncomplicated appendicitis from key stakeholder groups' perspectives (including paediatric surgeons, general surgeons, patients [12-18 years old], and parents of children who have had acute uncomplicated appendicitis).
3. To compare and contrast paediatric acute uncomplicated appendicitis treatment outcomes prioritised by key stakeholder groups (as detailed above).
4. To achieve consensus between key stakeholder groups on a COS to evaluate overall success of treatment for acute uncomplicated appendicitis in children.

Design

COS development will entail four key stages:

1. A systematic review to identify previously reported acute uncomplicated appendicitis treatment outcomes.
2. Assembly of stakeholder panels.
3. A three-phase online Delphi process.
4. Consensus meeting.

The CONTRACT study, as part of the feasibility study, will involve in-depth qualitative research with patients, parents, and caregivers. This qualitative research will be used at each stage of the process to inform the project development.

Systematic review

The COMET Initiative recommend the use of systematic reviews in informing the first phase of the Delphi process.[12] Two recent systematic reviews will be used to inform the initial list of potential outcomes to be considered for the COS. The first review identified outcomes used in studies of paediatric appendicitis[13] and the second review aimed to determine safety and efficacy of non-operative treatment for acute appendicitis.[8] We have drawn from these reviews and conducted an updated literature search to identify outcomes that can be used to develop an initial list of outcomes. Identified outcomes were combined with closely similar outcomes from the operative treatment systematic review[13] to develop an initial list of outcomes (Appendix 1). Appendix 1 describes the eligibility criteria for inclusion of papers. All relevant articles will therefore be included that reported any non-operative treatment regimen for acute uncomplicated appendicitis in children with or without a comparative group of children undergoing surgical treatment.

Finalising and appropriate wording of initial outcomes

The systematic review will inform an initial list of treatment outcomes. To inform and support the CONTRACT study, a Study Specific Advisory Group (SSAG) has been assembled, comprising of 15-20 young people and parents. Young people recruited are children who have had appendicitis or children from the existing Clinical Research Network (CRN) (Children) Young Persons Advisory Groups (YPAGs). Parents are parents of children who have had appendicitis. A SSAG meeting will be held to present the initial list of treatment outcomes, to inform the addition and wording of outcomes, and to ensure outcomes are appropriately presented. Three versions of the stakeholder-facing materials will be developed for all rounds of the Delphi process, for each stakeholder panel (clinicians, young people and parents), using appropriate language identified and agreed by the SSAG. The CONTRACT qualitative study will also inform the initial outcomes.

Stakeholder panel assembly - identification and recruitment

https://mc.manuscriptcentral.com/bmjpo

Confidential: For Review Only

For the COS to be meaningful and relevant to those involved in the treatment of acute appendicitis, the COS needs to reflect the views of patients who have been treated for acute appendicitis, their parents, and relevant clinicians. As these groups may have different priorities that could obstruct reaching consensus on a COS, the stakeholders will be separated into three panels, which we intend to be equally weighted: (1) patients; (2) parents; (3) paediatric surgeons and general surgeons. Initially, potential members of each stakeholder panel known to the research team will be invited to participate and we will develop strategies to identify further experts (e.g. use of membership lists of national organisations such as the British Association of Paediatric Surgeons) (Table 1).

Potential members of stakeholder groups will be invited to participate. This initial contact will explain the nature of the study and why they have been identified as a potential participants. It will contain a plain language summary of the study aims and procedures, emphasising the importance of commitment to the panel. The wording of this initial contact will be tailored to meet the panel category. It will also contain a link to an online form to enable participants to express their interest in participation in the study and to provide further information on their experience of the treatment of acute appendicitis. We will ask participants to commit to completing three rounds of questionnaires anticipated to take approximately 10 minutes each to complete.

The process of invitation and enrolment will continue until the optimal number of stakeholders have expressed an interest to participate (with at least ten in each panel). There is no consensus on the optimal sample size for a Delphi study; recruitment will therefore be based on previous Delphi studies.[14] We will aim to achieve 75-100 participants in the first round of the Delphi with at least as many parents/children as clinicians. We will aim to send the first questionnaire to all participants on the same day they each confirm their desire to participate.[15] Participants will be sent a link to a customised online database hosted on a secure server, from which they can access and complete phase one questionnaire of the Delphi process. To limit attrition appropriate procedures will be completed,[14 16] including reminder emails.

Table 1. List of key stakeholders

Stakeholder group	Who are they and how might they be approached about COS development?
Patients and parents	Patients and parents will be identified by clinical teams from the three sites that are participating in the CONTRACT study and also at further participant identification sites. These will be children aged 12-18 years who have been treated for acute uncomplicated appendicitis in the preceding 12-24 months (patients) and parents of children (any age) who have been treated for acute uncomplicated appendicitis in the preceding 12-24 months. These families may or may not have participated in CONTRACT. The patient and parent panels will specifically include children and parents treated initially by non-operative management as well as those treated operatively.
Paediatric surgeons	All practising consultant paediatric surgeons in the United Kingdom who treat children with acute uncomplicated appendicitis will be considered potential participants. They will be invited to participate through the mailing list of the British Association of Paediatric Surgeons and through personal contacts of the investigators.
General surgeons	Adult general surgeons in the UK who regularly treat children with acute uncomplicated appendicitis will be considered potential participants. This will include those identified as having an interest in the treatment of children.

Confidential: For Review Only

Confidential: For Review Only

Definition of consensus

During the process, participants will be asked to provide a score for each outcome (including those they have added themselves) using the Grading of Recommendations, Assessment, Development and Evaluations (GRADE) scale, which is recommended by the COMET initiative.[1] The scale will be presented in the format 1 to 9, with 1 to 3 labelled 'not important', 4 to 6 labelled 'important but not critical' and 7 to 9 labelled 'critical'.[17] 'Consensus in' will be defined as ≥ 70% of participants rating the outcome 7–9, and < 15% rating it as 1–3. Outcomes will defined as "consensus out" if > 70% participants rate it 1–3 and < 15% rate it 7–9. Outcomes not meeting these definitions will be classified as 'no consensus'.

Delphi process: phase one data collection

A customised online system will be developed to conduct a three-phase Delphi process run in parallel across the stakeholder panels. Participants will be presented with the initial list of outcomes, grouped by domains. As described, the initial list will comprise outcomes identified in our recent systematic review,[13] updated review of the literature (Appendix 1) and any additional outcomes identified during qualitative interviews with key stakeholders. There will also be an option for experts to add further outcomes but these outcomes will not be scored in phase one.

Surgeon participants will be asked the key question 'How important do you consider the following outcomes to be when considering which treatment to offer children with uncomplicated acute appendicitis?' A similar question will be posed to patient and parent stakeholder panels, with the wording altered as necessary based on our SSAG input.

Participants will be asked to complete each round of the Delphi exercise within 3 weeks and will be sent reminders via email when they have 1 week and 48 hours remaining to complete the survey. Participants who have not completed the questionnaire within 4 weeks of being requested to complete the questionnaire will be deemed not to have completed that phase.

Delphi process: phase one data analysis

The number of participants who were invited to participate in the first phase of the Delphi will be recorded, as well as the response rate from each stakeholder group. Outcomes will be analysed separately for each panel and descriptive statistics will be calculated for each. All outcomes will be carried forward to the second phase.

Additional outcomes provided by participants will be reviewed by two members of the COS team to ensure they represent new outcomes and will be included and carried forward to phase two so long as they were proposed by at least two participants.

Delphi process: phase two data collection

Participants who completed phase one will be invited to participate in phase two. Participants will be individually presented with their own scores, the distribution of scores for each outcome from their stakeholder group in phase one, and will subsequently be asked to rescore each outcome. Participants will be asked to score any new outcomes identified in phase one.

Delphi process: phase two data analysis

The data analysis process described for phase one will be repeated. Bias from loss of experts between phases will be assessed. Any outcomes that meet the criteria of 'consensus out' will be removed from the outcomes list prior to phase three. All other outcomes from phase two will be carried forward to phase three.

Delphi process: phase three data collection

Participants who completed phases one and two will be invited to participate in phase three. The data collection process described for phase two will be repeated, however, participants will also be shown scores for their own stakeholder panel and separately for each other panel. This will allow clinicians to take into account the views of children and parents and vice-versa before rescoring the outcomes.[18] Participants will be asked to identify the one outcome which they believe is the most important for informing their treatment choice, and if they cannot identify a single outcome, a combination of essential outcomes.

Delphi process: phase three data analysis

The data analysis process described for phase two will be repeated. All outcomes from phase three will be carried forward to the consensus meeting

Final consensus meeting

The aim of the consensus meeting is to ratify outcomes where consensus ('in' or 'out') has been achieved, discuss outcomes where consensus could not be achieved and to finalise the COS. All participants who have completed all 3 rounds of the Delphi exercise will be invited to attend the consensus meeting. We will aim to have a minimum of 40 stakeholders confirm their attendance with equally weighted panels and disciplines. Representatives from each stakeholder panel will be required in order for the consensus meeting to be quorate.

During the meeting, stakeholders will be provided with an overview of the results of phase three of the Delphi exercise including presentation of each outcome scored, how it was scored by each stakeholder panel and its consensus status. Following moderated discussion, each outcome will be anonymously re-scored using the same scoring system as the Delphi process. For those outcomes for which 'no consensus' was achieved across all stakeholder panels at the end of the Delphi exercise, and for which consensus was achieved in at least one but not all stakeholder groups, further discussion will take place, following which attendees will be asked to score each outcome anonymously. Following re-scoring at the consensus meeting, outcomes reaching 'consensus in' will be included in the finalised COS. All others will be excluded.

Finalising the COS

The Outcome Measures in Rheumatology (OMERACT) initiative provides a comprehensive conceptual framework and recommends a process to develop COS development that is a useful tool for various areas of healthcare.[19] In developing the current COS, we will draw on this framework, which comprises of three core domains (Death, Life Impact and Pathophysiological Manifestations) and one strongly recommended domain (Resource Use). The framework recommends that at least one applicable measurement instrument for each core domain should be used to formulate a COS. It also recommends inclusion of 'adverse events'. Overall, we aim to achieve a manageable COS with a maximum of approximately 10 outcomes.

Data management

Experts will enter data directly into the customised database when they complete each questionnaire at each phase of the Delphi process. Anonymised data will be stored securely and will

https://mc.manuscriptcentral.com/bmjpo

Confidential: For Review Only

be managed as per standard operating procedures. Only selected members of the research team will have access to the data.

ETHICS AND DISSEMINATION

Full ethical approval for CONTRACT was granted by South Central - Hampshire A Research Ethics Committee (REC ref: 16/SC/0596) in November 2016. The finalised COS will be disseminated via publication, events and the COMET Initiative website (http://www.comet-initiative.org/).

FULL REFERENCES

1. St Peter SD, Tsao K, Spilde TL, et al. Single daily dosing ceftriaxone and metronidazole vs standard triple antibiotic regimen for perforated appendicitis in children: a prospective randomized trial. Journal of pediatric surgery 2008;**43**(6):981-5.
2. Blomqvist PG, Andersson REB, Granath F, et al. Mortality After Appendectomy in Sweden, 1987–1996. Annals of Surgery 2001;**233**(4):455-60.
3. Styrud J, Eriksson S, Nilsson I, et al. Appendectomy versus antibiotic treatment in acute appendicitis. a prospective multicenter randomized controlled trial. World journal of surgery 2006;**30**(6):1033-7.
4. Hansson J, Korner U, Khorram-Manesh A, et al. Randomized clinical trial of antibiotic therapy versus appendicectomy as primary treatment of acute appendicitis in unselected patients. The British journal of surgery 2009;**96**(5):473-81.
5. Vons C, Barry C, Maitre S, et al. Amoxicillin plus clavulanic acid versus appendicectomy for treatment of acute uncomplicated appendicitis: an open-label, non-inferiority, randomised controlled trial. Lancet 2011;**377**(9777):1573-9.
6. Svensson JF, Patkova B, Almstrom M, et al. Nonoperative treatment with antibiotics versus surgery for acute nonperforated appendicitis in children: a pilot randomized controlled trial. Ann Surg 2015;**261**(1):67-71.
7. Minneci PC, Sulkowski JP, Nacion KM, et al. Feasibility of a nonoperative management strategy for uncomplicated acute appendicitis in children. Journal of the American College of Surgeons 2014;**219**(2):272-9.
8. Georgiou R, Eaton S, Stanton MP, et al. Efficacy and Safety of Nonoperative Treatment for Acute Appendicitis: A Meta-analysis. Pediatrics 2017;**139**(3).
9. Sinha IP, Altman DG, Beresford MW, et al. Standard 5: Selection, Measurement, and Reporting of Outcomes in Clinical Trials in Children. Pediatrics 2012;**129**(Supplement 3):S146.
10. Williamson P, Altman D, Blazeby J, et al. Driving up the quality and relevance of research through the use of agreed core outcomes. Journal of health services research & policy 2012;**17**(1):1-2.
11. Gargon E, Gurung B, Medley N, et al. Choosing important health outcomes for comparative effectiveness research: a systematic review. PloS one 2014;**9**(6):e99111.
12. Williamson PR, Altman DG, Blazeby JM, et al. Developing core outcome sets for clinical trials: issues to consider. Trials 2012;**13**:132.
13. Hall NJ, Kapadia MZ, Eaton S, et al. Outcome reporting in randomised controlled trials and meta-analyses of appendicitis treatments in children: a systematic review. Trials 2015;**16**:275.
14. Sinha IP, Smyth RL, Williamson PR. Using the Delphi technique to determine which outcomes to measure in clinical trials: recommendations for the future based on a systematic review of existing studies. PLoS Med 2011;**8**(1):e1000393.
15. Delbecq AL, Van de Ven AH, Gustafson DH. *Group techniques for program planning: A guide to nominal group and Delphi processes*. Illinois: Scott, Foresman and Company, 1975.
16. Young B, Bagley H. Including patients in core outcome set development: issues to consider based on three workshops with around 100 international delegates. Research Involvement and Engagement 2016;**2**(1):25.

https://mc.manuscriptcentral.com/bmjpo

Confidential: For Review Only

17. Guyatt GH, Oxman AD, Kunz R, et al. GRADE guidelines: 2. Framing the question and deciding on important outcomes. J Clin Epidemiol 2011;**64**(4):395-400.
18. Harman NL, Bruce IA, Kirkham JJ, Tierney S, Callery P, O'Brien K, Bennett AM, Chorbachi R, Hall PN, Harding-Bell A. The importance of integration of stakeholder views in core outcome set development: Otitis media with effusion in children with cleft palate. PLoS One. 2015;10(6):e0129514.
19. Boers M, Kirwan JR, Wells G, et al. Developing Core Outcome Measurement Sets for Clinical Trials: OMERACT Filter 2.0. Journal of Clinical Epidemiology 2014;**67**(7):745-53.

AUTHORS' CONTRIBUTIONS

FS, SE and NH developed a first draft of the protocol. All authors advised on the development of the protocol and contributed towards the revision of the protocol for final submission.

FUNDING STATEMENT

The CONTRACT study is supported by the United Kingdom National Institute for Health Research Health Technology Assessment Programme (Grant number: 14/192/90 http://www.nets.nihr.ac.uk/projects/hta/1419290).

COMPETING INTERESTS STATEMENT

The authors report no competing interests.

ACKNOWLEDGEMENTS

All authors gratefully acknowledge funding from the National Institute for Health Research Health Technology Assessment Programme the CONTRACT trial, including the COS Development. SE gratefully acknowledges funding from Great Ormond Street Children's Charity.

Confidential: For Review Only

APPENDIX 1. Systematic review of published outcomes of conservative treatment of acute appendicitis in children

All papers identified by our systematic review of conservative treatment[1] were included, and the literature search in that paper repeated to identify any further papers published between 2016 and April 2017.

Confidential: For Review Only

Table 1. Characteristics of included studies

Study	Publication Year	Region	Study Type	Sample size (intention to treat non-operatively)	Age of included population
Abes[2]	2007	Antibiotics	Retrospective	Middle East	16
Armstrong[3]	2014	Antibiotics vs. appendectomy	Retrospective	North America	12
Caruso[4]	2017	Antibiotics vs. appendectomy	Retrospective	Europe	197
Chen[5]	2016	Antibiotic	Retrospective	Asia	125
Gorter[6]	2015	Antibiotics	Prospective	Europe	25
Hartwich[7]	2016	Antibiotics	Prospective	North America	24
Jimbo[8]	2016	Antibiotics	Retrospective	Asia	71
Kaneko[9]	2004	Antibiotic	Prospective	Asia	91
Koike[10]	2014	Antibiotics	Retrospective	Asia	134
Mahida[11]	2016	Antibiotics	Prospective	North America	5
Minecci[12]	2016	Antibiotics	Prospective	North America	37
Mudri[13]	2017	Antibiotics and appendectomy	Retrospective	North America	26
Steiner[14]	2015	Antibiotics	Retrospective	Middle-east	45
Steiner[15]	2017	Antibiotics	Retrospective	Middle-east	197
Svensson[16]	2015	Antibiotics and appendectomy	Prospective RCT	Europe	24
Tanaka[17]	2015	Antibiotics and appendectomy	Retrospective	Japan	78

Outcomes from these papers were then mapped, where possible, to the outcomes identified in our previous systematic review of outcomes of appendicitis in children[18].

Confidential: For Review Only

Table 2. Outcomes mapped to those previously described[18]

	Abes	Armstrong	Caruso	Chen	Gorter	Hartwich	Jimbo	Kaneko	Koike	Mahida	Minecci	Mudri	Steiner 2015	Steiner 2017	Svensson	Tanaka
**Wound Infection**			x		x								x			
**Intra-abdominal abscess**			x	x	x							x				
**(Adhesive) obstruction**			x													
**Complication of antibiotics or treatment intervention**					x											
**Major/minor complications**		x														
**Non-infectious wound complications**																
**Re-admission to hospital**		x	x		x	X					x	x				
**Other infectious complications**		x		x								x				
**Interventional radiology procedure**																
**Need for operation/re-operation**			x		x	x	x	x		x	x	x	x	x	x	x
**Conversion laparoscopic to open surgery**																
**Recurrent appendicitis**	x	x			x	x	x	x	x	x	x	x	x	x	x	x
**Other complications**			X		x											
**Bacterial isolates**																
**Post-treatment fever**													x	x		
**Measure of recovery of gastro-intestinal function**							x									
**Blood markers (WCC/CRP)**			x										x	x		
**Duration of abdominal drainage**																
**Validated pain score**																
**Other pain assessment**	x		x										x		x	

Confidential: For Review Only

**Time away from normal activities or school**											x					
**Recovery to full activity/sport**																
**Cosmesis**																
**Time to ambulation**																
**Other PROM**																x
**Duration of home healthcare**																x
**Paediatric quality of life assessment**						x					x					
**Parental quality of life assessment**						x					x					
**Hospital length of stay**		x	x	x			x		x		x	x	x	x	x	x
**Duration of surgery/analgesia**							x									
**Total charges**						x			x		x	x			x	
**Duration of antibiotics**														x		
**Narcotic/analgesia doses**																
**Healthcare visits**		x														
**Post-treatment imaging**	x		x					x					x	x		x
**Cost effectiveness**						x	x					x				
**Death**																

No completely new outcomes were identified using this search. However, perforation is considered as a major complication, and it may be required to elevate this to the level of a separate outcome in the Core outcome set.

Confidential: For Review Only

References

1. Georgiou R, Eaton S, Stanton MP, et al. Efficacy and Safety of Nonoperative Treatment for Acute Appendicitis: A Meta-analysis. Pediatrics 2017;**139**(3).
2. Abes M, Petik B, Kazil S. Nonoperative treatment of acute appendicitis in children. J Pediatr Surg 2007;**42**(8):1439-42.
3. Armstrong J, Merritt N, Jones S, et al. Non-operative management of early, acute appendicitis in children: Is it safe and effective? Journal of Pediatric Surgery 2014;**http://dx.doi.org/10.1016/j.jpedsurg.2014.02.071**.
4. Caruso AM, Pane A, Garau R, et al. Acute appendicitis in children: not only surgical treatment. J Pediatr Surg 2017;**52**(3):444-48.
5. Chen CL, Chao HC, Kong MS, et al. Risk Factors for Prolonged Hospitalization in Pediatric Appendicitis Patients with Medical Treatment. Pediatrics and neonatology 2017;**58**(3):223-28.
6. Gorter RR, van der Lee JH, Cense HA, et al. Initial antibiotic treatment for acute simple appendicitis in children is safe: Short-term results from a multicenter, prospective cohort study. Surgery 2015;**157**(5):916-23.
7. Hartwich J, Luks FI, Watson-Smith D, et al. Nonoperative treatment of acute appendicitis in children: A feasibility study. J Pediatr Surg 2016;**51**(1):111-6.
8. Jimbo K, Takeda M, Miyata E, et al. Is a pediatrician performed gray scale ultrasonography with power Doppler study safe and effective for triaging acute non-perforated appendicitis for conservative management? J Pediatr Surg 2016;**51**(12):1952-56.
9. Kaneko K, Tsuda M. Ultrasound-based decision making in the treatment of acute appendicitis in children. J Pediatr Surg 2004;**39**(9):1316-20.
10. Koike Y, Uchida K, Matsushita K, et al. Intraluminal appendiceal fluid is a predictive factor for recurrent appendicitis after initial successful non-operative management of uncomplicated appendicitis in pediatric patients. Journal of Pediatric Surgery 2014;**49**(7):1116-21.
11. Mahida JB, Lodwick DL, Nacion KM, et al. High failure rate of nonoperative management of acute appendicitis with an appendicolith in children. J Pediatr Surg 2016;**51**(6):908-11.
12. Minneci PC, Mahida JB, Lodwick DL, et al. Effectiveness of Patient Choice in Nonoperative vs Surgical Management of Pediatric Uncomplicated Acute Appendicitis. JAMA Surg 2016;**151**(5):408-15.
13. Mudri M, Coriolano K, Butter A. Cost analysis of nonoperative management of acute appendicitis in children. J Pediatr Surg 2017.
14. Steiner Z, Buklan G, Stackievicz R, et al. A role for conservative antibiotic treatment in early appendicitis in children. J Pediatr Surg 2015;**50**(9):1566-8.
15. Steiner Z, Buklan G, Stackievicz R, et al. Conservative treatment in uncomplicated acute appendicitis: reassessment of practice safety. Eur J Pediatr 2017;**176**(4):521-27.
16. Svensson JF, Patkova B, Almstrom M, et al. Nonoperative treatment with antibiotics versus surgery for acute nonperforated appendicitis in children: a pilot randomized controlled trial. Ann Surg 2015;**261**(1):67-71.
17. Tanaka Y, Uchida H, Kawashima H, et al. Long-term outcomes of operative versus nonoperative treatment for uncomplicated appendicitis. J Pediatr Surg 2015;**50**(11):1893-7.
18. Hall NJ, Kapadia MZ, Eaton S, et al. Outcome reporting in randomised controlled trials and meta-analyses of appendicitis treatments in children: a systematic review. Trials 2015;**16**:275.

Confidential: For Review Only

BMJ Paediatrics Open

Confidential: For Review Only

Development of a core outcome set to determine the overall treatment success of acute uncomplicated appendicitis in children: a study protocol

Journal:	*BMJ Paediatrics Open*
Manuscript ID	bmjpo-2017-000151.R1
Article Type:	Protocol
Date Submitted by the Author:	20-Jul-2017
Complete List of Authors:	Sherratt, Frances; University of Liverpool, Department of Psychological Sciences<br>Eaton, Simon; UCL Institute of Child Health, Developmental Biology and Cancer Programme<br>Walker, Erin; Great Ormond Street Hospital For Children NHS Trust<br>Beasant, Lucy; University of Bristol School of Social and Community Medicine, Centre for Child and Adolescent Health<br>Blazeby, Jane; University of Bristol School of Social and Community Medicine, Centre for Surgical Research<br>Young, Bridget; University of Liverpool, Department of Psychological Sciences<br>Crawley, Esther; University of Bristol School of Social and Community Medicine, Centre for Child and Adolescent Health<br>Wood, Wendy; University of Southampton, NIHR Research Design Services South Central<br>Hall, Nigel; University of Southampton, University Surgery Unit, Faculty of Medicine
Keywords:	Paediatric Practice, Paediatric Surgery, Measurement

SCHOLARONE™
Manuscripts

Development of a core outcome set to determine the overall treatment success of acute uncomplicated appendicitis in children: a study protocol

Frances C Sherratt[1], Simon Eaton[2], Erin Walker[3], Lucy Beasant[4], Jane M Blazeby[5], Bridget Young[1], Esther Crawley[4], Wendy W Wood[6], Nigel J Hall[7]

1. Institute of Psychology, University Of Liverpool, Liverpool, UK

2. Developmental Biology and Cancer Programme, UCL Great Ormond Street Institute of Child Health, London, UK

3. Great Ormond Street Hospital NHS Foundation Trust, London, UK

4. Centre for Child and Adolescent Health, School of Social and Community Medicine, University of Bristol, Bristol, UK

5. Centre for Surgical Research, School of Social & Community Medicine, University of Bristol, Bristol, UK

6. Research Design Services South Central, University of Southampton, Southampton, UK

7. University Surgery Unit, Faculty of Medicine, University of Southampton, Southampton, UK

Corresponding author:

Dr Frances C Sherratt

Department of Psychological Sciences, University of Liverpool, Whelan Building, Liverpool, L69 3GB

E-mail: sherratt@liverpool.ac.uk

Tel: +44151 795 0347

Word count, excluding title page, abstract, references, figures and tables: 2,496

Keywords: appendicectomy, appendectomy, appendicitis, antibiotics, children, paediatric, core outcomes, core outcome set, Delphi, consensus meeting

Confidential: For Review Only

ABSTRACT

Introduction

In recent years, there has been growing interest in alternatives to appendicectomy. In particular, non-operative treatment of appendicitis, with antibiotics alone, has been proposed as a potential treatment. A small number of randomised controlled trials (RCTs) in adults and, more recently, children suggest that antibiotic treatment may be a valid alternative to appendicectomy. However, there is currently insufficient data to justify its widespread use. Prior to performing further efficacy studies of the treatment of appendicitis in children, it is imperative to identify the most relevant outcome measures for inclusion in the design of comparative studies. This is of particular importance when evaluating a novel treatment approach since the outcomes of importance may differ from those commonly reported with traditional therapies.

A review of the relevant literature and electronic resources failed to identify a Core Outcome Set (COS) for children with appendicitis. We aim to define a COS for the measurement of treatment interventions in children (< 18 years) with acute appendicitis

Methods and analysis

This project will entail: (i) a systematic review to identify previously reported acute uncomplicated appendicitis treatment outcomes; (ii) assembly of stakeholder panels (paediatric and adult surgeons, patients and parents); (iii) a three stage Delphi process, and; (iv) a final consensus meeting to complete the COS.

Ethics and Registration

COS development is part of CONservative TReatment of Appendicitis in Children - a randomised controlled Trial (Feasibility) - CONTRACT study, for which full ethical approval for CONTRACT has been granted. The COS development study is registered with the COMET initiative in May 2017 (http://www.comet-initiative.org/studies/details/987).

KEY MESSAGES

What is known about the subject?

- Traditionally, appendicectomy has been the gold standard treatment for acute appendicitis in children but there has been increased interest in non-operative treatment (with antibiotics).
- Core outcome sets are developed and adopted to avoid inconsistencies in outcome selection, measurement and reporting that may otherwise exist.
- There is currently no core outcome set for the measurement of effectiveness of treatment interventions in children with acute uncomplicated appendicitis.

What this study adds:

- This project will involve defining a core outcome set for the measurement of effectiveness of treatment interventions in children with acute uncomplicated appendicitis.
- Considering outcomes of importance to patients, parents of patients and health professionals is crucial for paediatric appendicitis research to be meaningful and relevant.

Confidential: For Review Only

BACKGROUND

A lack of knowledge and understanding regarding which outcomes are important to patients and clinicians may result in important outcomes being omitted from clinical trials. Differences in outcome selection and reporting between studies and how outcomes are defined and measured also make it difficult, sometimes impossible, to synthesise results of studies (e.g. meta-analysis) and apply them in a meaningful way. To address these problems, core outcome sets (COS) have been proposed as a means of standardizing outcome selection, measurement and reporting in healthcare research and in clinical trials in particular.[1 2] The development of a COS and its adoption by researchers is intended to help avoid inconsistencies in outcome selection, measurement and reporting that may otherwise exist. If trials do not adopt an established COS they risk selecting suboptimal outcomes and are unlikely to contribute usable information.[3]

For children with acute uncomplicated appendicitis, appendicectomy has traditionally been the gold standard treatment but there has been growing interest in alternatives to appendicectomy. In particular, non-operative treatment of appendicitis, with antibiotics has been proposed as a potential treatment. A small number of randomised controlled trials (RCTs) in adults[4-6] and, more recently, RCTs and prospective preference studies in children[7 8] suggest that antibiotic treatment may be a valid alternative to appendicectomy.[9] However, there is currently insufficient data to justify its widespread use. Prior to performing further efficacy studies of the treatment of appendicitis in children, it is imperative to identify the most relevant outcomes for inclusion in the design of comparative studies. This is of particular importance when evaluating a novel treatment approach since the outcomes of importance may differ from those commonly reported with traditional therapies. A review of the relevant literature and electronic resources failed to identify a COS for children with appendicitis.[10] Furthermore a wide range of outcomes were reported and a range of different primary outcomes were used across studies.

In order to advance our understanding of which outcomes are important and to fulfil an unmet need in our future research programme, the aim of this study is to develop a COS for the measurement of effectiveness of treatment interventions in children (< 18 years) with acute uncomplicated appendicitis.

METHODS

The COS development is a component of a wider project, CONservative TReatment of Appendicitis in Children - a randomised controlled Trial (Feasibility) - CONTRACT study (http://www.nets.nihr.ac.uk/projects/hta/1419290). The COS development study was registered with the COMET initiative in May 2017 (http://www.comet-initiative.org/studies/details/987).

Scope of the COS

The COS is intended to be used to evaluate the overall success of operative and non-operative treatment among children who are assigned a clinical and/or radiological diagnosis of acute uncomplicated appendicitis. The COS will include outcome measures identified as important within 12 months of treatment initiation and longer term outcomes if applicable. The COS focuses specifically on treatment of acute uncomplicated appendicitis; the treatment of perforated appendicitis (with or without abscess) and appendix mass is outside the scope of this COS. The key objectives of the study are:

1. To determine which outcomes have previously been reported in studies comparing treatments for acute uncomplicated appendicitis in children.

https://mc.manuscriptcentral.com/bmjpo

Confidential: For Review Only

2. To prioritise treatment outcomes of children with acute uncomplicated appendicitis from key stakeholder groups' perspectives (including paediatric surgeons, general surgeons, patients [12-18 years old], and parents of children who have had acute uncomplicated appendicitis).
3. To compare and contrast paediatric acute uncomplicated appendicitis treatment outcomes prioritised by key stakeholder groups (as detailed above).
4. To achieve consensus between key stakeholder groups on a COS to evaluate overall success of treatment for acute uncomplicated appendicitis in children.

Design

COS development will entail four key stages:

1. A systematic review to identify previously reported acute uncomplicated appendicitis treatment outcomes.
2. Assembly of stakeholder panels.
3. A three-phase online Delphi process.
4. Consensus meeting.

The CONTRACT study, as part of the feasibility study, will involve in-depth qualitative research. Interviews will be conducted with patients, parents and caregivers to explore experiences of treatment for appendicitis. We will explore families' perceptions of meaningful and important outcomes, which will help to optimise COS participants' project understanding and engagement throughout the COS development.

Systematic review

The COMET Initiative recommend the use of systematic reviews in informing the first phase of the Delphi process.[11] Two recent systematic reviews will be used to inform the initial list of potential outcomes to be considered for the COS. The first review identified outcomes used in studies of paediatric appendicitis[10] and the second review aimed to determine safety and efficacy of non-operative treatment for acute appendicitis.[9] Identified outcomes were combined with closely similar outcomes from the operative treatment systematic review[10] to develop an initial list of outcomes (Appendix 1). Appendix 1 describes the eligibility criteria for inclusion of papers. All relevant articles will therefore be included that reported any non-operative treatment regimen for acute uncomplicated appendicitis in children with or without a comparative group of children undergoing surgical treatment. All outcomes identified through these reviews and an updated literature search will inform the initial list of outcomes and will be considered by the stakeholder groups as part of the Delphi process.

Finalising and appropriate wording of initial outcomes

To inform and support the CONTRACT study, a Study Specific Advisory Group (SSAG) has been assembled, comprising of 15-20 young people and parents. Young people recruited are children who have had appendicitis or children from the existing Clinical Research Network (Children) Young Persons Advisory Groups. Parents are parents of children who have had appendicitis. A SSAG meeting will be held to present the initial list of treatment outcomes, to inform the addition and wording of outcomes, and to ensure outcomes are appropriately presented. Three versions of the stakeholder-facing materials will be developed for all rounds of the Delphi process, for each stakeholder panel (clinicians, young people and parents), using appropriate language identified and agreed by the SSAG. The CONTRACT qualitative study will also inform the initial outcomes.

https://mc.manuscriptcentral.com/bmjpo

Confidential: For Review Only

Stakeholder panel assembly - identification and recruitment

For the COS to be meaningful and relevant to those involved in the treatment of acute appendicitis, the COS needs to reflect the views of patients who have been treated for acute appendicitis, their parents, and relevant clinicians. As these groups may have different priorities that could obstruct reaching consensus on a COS, the stakeholders will be separated into three panels, which we intend to be equally weighted: (1) patients; (2) parents; (3) paediatric surgeons and general surgeons. Initially, potential members of each stakeholder panel known to the research team will be invited to participate and we will develop strategies to identify further experts (see Table 1).

Initial contact with potential participants will explain the study and why they have been identified as a potential participants. It will contain a plain language summary of the study aims and procedures, emphasising the importance of commitment to the panel. The wording of this initial contact will be tailored to meet the panel category. It will also contain a link to an online form to enable participants to express their interest in participation in the study and to provide further information on their experience of the treatment of acute appendicitis. We will ask participants to commit to completing three rounds of questionnaires anticipated to take approximately 10 minutes each to complete.

The process of invitation and enrolment will continue until the optimal number of stakeholders have expressed an interest to participate (with at least ten in each panel). There is no consensus on the optimal sample size for a Delphi study; recruitment will therefore be based on previous Delphi studies.[12] We will aim to achieve 75-100 participants in the first round of the Delphi with at least as many parents/children as clinicians. Efforts will be made to invite a diverse range of participants to each stakeholder group. We will aim to send the first questionnaire to all participants on the same day they each confirm their desire to participate.[13] Participants will be sent a link to a customised online database hosted on a secure server, from which they can access and complete phase one questionnaire of the Delphi process. To limit attrition, appropriate procedures will be completed,[12 14] including reminder emails.

Participation in this COS development process will be limited to surgeons, patients and parents from the UK. We will not recruit paediatric or general surgeons from outside the UK because the treatment pathway for appendicitis in the UK differs to that in other countries. Furthermore, if we were to recruit surgeons from outside the UK, we would also need to recruit patients and parents from outside the UK to avoid bias; this would become increasingly challenging logistically.

Table 1. Core outcome set stakeholder groups and methods of approaching potential participants

Stakeholder group	Selection criteria	Method of approach
Patients and parents	- Patients aged 12-18 years who have been treated for acute uncomplicated appendicitis in the preceding 12-24 months.<br>- Parents of children (aged 5-18 years) who have been treated for acute uncomplicated appendicitis in the preceding 12-24 months.<br>Families may or may not have participated in CONTRACT.<br>- Patient and parent panels will specifically include children and parents treated initially by non-operative management as well as those treated operatively.	- Invited to participate via clinical teams from the three sites that are participating in the CONTRACT study.<br>- Identified to participate via further participant identification sites.
Paediatric	- All practising consultant paediatric surgeons in the United	- Invited to

https://mc.manuscriptcentral.com/bmjpo

Confidential: For Review Only

surgeons	Kingdom (UK) who treat children with acute uncomplicated appendicitis will be considered potential participants.	participate via the mailing list of the British Association of Paediatric Surgeons and through personal contacts of the investigators.
General surgeons	- Adult general surgeons in the UK who regularly treat children with acute uncomplicated appendicitis will be considered potential participants. This will include those identified as having an interest in the treatment of children.	- Invited to participate via existing personal contacts and through regional paediatric surgical networks within the UK.

Confidential: For Review Only

Definition of consensus

During the process, participants will be asked to score each outcome using the Grading of Recommendations, Assessment, Development and Evaluations (GRADE) scale, which is recommended by the COMET initiative.[15] The scale will be presented in the format 1 to 9, with 1 to 3 labelled 'not important', 4 to 6 labelled 'important but not critical' and 7 to 9 labelled 'critical'.[16] 'Consensus in' will be defined as ≥ 70% of participants rating the outcome 7–9, and < 15% rating it as 1–3. Outcomes will defined as "consensus out" if > 70% participants rate it 1–3 and < 15% rate it 7–9. Outcomes not meeting these definitions will be classified as 'no consensus'.

Delphi process: phase one data collection

A customised online data system (developed by the National Perinatal Epidemiology Unit [University of Oxford, UK]) will be used to conduct a three-phase Delphi process run in parallel across the stakeholder panels. Participants will be presented with the initial list of outcomes, grouped by domains. As described, the initial list will comprise outcomes identified in our recent systematic review,[10] updated review of the literature (Appendix 1) and any additional outcomes identified during qualitative interviews with key stakeholders. There will also be an option for experts to add further outcomes but these outcomes will not be scored in phase one.

Surgeon participants will be asked the key question 'How important do you consider the following outcomes to be when considering which treatment to offer children with uncomplicated acute appendicitis?' A similar question will be posed to patient and parent stakeholder panels, with the wording altered as necessary based on our SSAG input.

Participants will be asked to complete each round of the Delphi exercise within 3 weeks and two subsequent reminder emails will be sent. Participants who have not completed the questionnaire within 4 weeks of being requested to complete the questionnaire will be deemed not to have completed that phase.

Delphi process: phase one data analysis

The number of participants who were invited to participate in phase one will be recorded and the response rate from each stakeholder group. Outcomes will be analysed separately for each panel and descriptive statistics will be calculated. All outcomes will be carried forward to phase two.

Additional outcomes provided by participants will be reviewed by two members of the COS team to ensure they represent new outcomes and will be included in phase two if they were proposed by at least two participants.

Delphi process: phase two data collection

Participants who completed phase one will be invited to participate in phase two. Participants will be individually presented with their own scores, the distribution of scores for each outcome from their stakeholder group in phase one, and will be asked to rescore each outcome. Participants will be asked to score any new outcomes identified in phase one.

Delphi process: phase two data analysis

The data analysis process described for phase one will be repeated. Bias from loss of experts between phases will be assessed. Any outcomes that meet the criteria of 'consensus out' will be removed from the outcomes list prior to phase three. All other outcomes from phase two will be carried forward to phase three.

Delphi process: phase three data collection

Participants who completed phases one and two will be invited to participate in phase three. The data collection process described for phase two will be repeated, however, participants will also be shown scores for their own stakeholder panel and separately for each other panel. This will allow participants to consider other stakeholder groups' views before rescoring the outcomes.[17] Participants will be asked to identify the one outcome which they believe is the most important for informing their treatment choice, and if they cannot identify a single outcome, a combination of essential outcomes.

Delphi process: phase three data analysis

The data analysis process described for phase two will be repeated. All outcomes from phase three will be carried forward to the consensus meeting.

Final consensus meeting

The aim of the consensus meeting is to ratify outcomes where consensus ('in' or 'out') has been achieved, discuss outcomes where consensus could not be achieved and to finalise the COS. All participants who have completed all 3 rounds of the Delphi exercise will be invited to attend the consensus meeting. We will aim to have a minimum of 40 stakeholders confirm their attendance with equally weighted panels and disciplines. Representatives from each stakeholder panel will be required in order for the consensus meeting to be quorate.

During the meeting, stakeholders will be provided with an overview of the results of phase three including presentation of each outcome scored, how it was scored by each stakeholder panel and its consensus status. Following moderated discussion, each outcome will be anonymously re-scored using the same scoring system as the Delphi process. For outcomes for which 'no consensus' was achieved across all stakeholder panels at the end of the Delphi exercise, and for which consensus was achieved in at least one but not all stakeholder groups, further discussion will take place, following which attendees will be asked to score each outcome anonymously. Following re-scoring

https://mc.manuscriptcentral.com/bmjpo

Confidential: For Review Only

at the consensus meeting, outcomes reaching 'consensus in' will be included in the finalised COS. All others will be excluded.

Finalising the COS

The Outcome Measures in Rheumatology (OMERACT) initiative provide a COS development framework that is a useful across various healthcare domains.[18] In developing the current COS, we will draw on this framework, which comprises of three core domains (Death, Life Impact and Pathophysiological Manifestations) and one strongly recommended domain (Resource Use). The framework recommends inclusion of at least one applicable measurement instrument for each core domain. It also recommends inclusion of 'adverse events'. Overall, we aim to achieve a manageable COS with a maximum of approximately 10 outcomes. Beyond the current project, further work may be undertaken to establish optimal methods of measuring each core outcome[18] and subsequent work may be undertaken to determine whether the UK-based COS is valid internationally.

Data management

Experts will enter data directly into the customised database when they complete each questionnaire at each phase of the Delphi process. Anonymised data will be stored securely and will be managed as per standard operating procedures. Only selected members of the research team will have access to the data.

ETHICS AND DISSEMINATION

Full ethical approval for CONTRACT was granted by South Central - Hampshire A Research Ethics Committee (REC ref: 16/SC/0596) in November 2016. The finalised COS will be disseminated via publication, events and the COMET Initiative website (http://www.comet-initiative.org/).

FULL REFERENCES

1. Sinha IP, Altman DG, Beresford MW, et al. Standard 5: Selection, Measurement, and Reporting of Outcomes in Clinical Trials in Children. *Pediatrics* 2012;129(Supplement 3):S146.
2. Williamson P, Altman D, Blazeby J, et al. Driving up the quality and relevance of research through the use of agreed core outcomes. *Journal of health services research & policy* 2012;17(1):1-2. doi: 10.1258/jhsrp.2011.011131 [published Online First: 2012/02/02]
3. Gargon E, Gurung B, Medley N, et al. Choosing important health outcomes for comparative effectiveness research: a systematic review. *PloS one* 2014;9(6):e99111. doi: 10.1371/journal.pone.0099111 [published Online First: 2014/06/17]
4. Styrud J, Eriksson S, Nilsson I, et al. Appendectomy versus antibiotic treatment in acute appendicitis. a prospective multicenter randomized controlled trial. *World J Surg* 2006;30(6):1033-37. doi: 10.1007/s00268-005-0304-6 [doi]
5. Hansson J, Korner U, Khorram-Manesh A, et al. Randomized clinical trial of antibiotic therapy versus appendicectomy as primary treatment of acute appendicitis in unselected patients. *Br J Surg* 2009;96(5):473-81. doi: 10.1002/bjs.6482 [doi]
6. Vons C, Barry C, Maitre S, et al. Amoxicillin plus clavulanic acid versus appendicectomy for treatment of acute uncomplicated appendicitis: an open-label, non-inferiority, randomised controlled trial. *Lancet* 2011;377(9777):1573-79. doi: S0140-6736(11)60410-8 [pii];10.1016/S0140-6736(11)60410-8 [doi]
7. Svensson JF, Patkova B, Almstrom M, et al. Nonoperative treatment with antibiotics versus surgery for acute nonperforated appendicitis in children: a pilot randomized controlled trial. *Ann Surg* 2015;261(1):67-71. doi: 10.1097/SLA.0000000000000835
8. Minneci PC, Sulkowski JP, Nacion KM, et al. Feasibility of a nonoperative management strategy for uncomplicated acute appendicitis in children. *Journal of the American College of Surgeons*

https://mc.manuscriptcentral.com/bmjpo

Confidential: For Review Only

2014;219(2):272-9. doi: 10.1016/j.jamcollsurg.2014.02.031 [published Online First: 2014/06/22]

9. Georgiou R, Eaton S, Stanton MP, et al. Efficacy and Safety of Nonoperative Treatment for Acute Appendicitis: A Meta-analysis. *Pediatrics* 2017;139(3) doi: 10.1542/peds.2016-3003
10. Hall NJ, Kapadia MZ, Eaton S, et al. Outcome reporting in randomised controlled trials and meta-analyses of appendicitis treatments in children: a systematic review. *Trials* 2015;16:275. doi: 10.1186/s13063-015-0783-1
11. Williamson PR, Altman DG, Blazeby JM, et al. Developing core outcome sets for clinical trials: issues to consider. *Trials* 2012;13:132. doi: 10.1186/1745-6215-13-132 [published Online First: 2012/08/08]
12. Sinha IP, Smyth RL, Williamson PR. Using the Delphi technique to determine which outcomes to measure in clinical trials: recommendations for the future based on a systematic review of existing studies. *PLoS Med* 2011;8(1):e1000393. doi: 10.1371/journal.pmed.1000393 [published Online First: 2011/02/02]
13. Delbecq AL, Van de Ven AH, Gustafson DH. Group techniques for program planning: A guide to nominal group and Delphi processes. Illinois: Scott, Foresman and Company 1975.
14. Young B, Bagley H. Including patients in core outcome set development: issues to consider based on three workshops with around 100 international delegates. *Research Involvement and Engagement* 2016;2(1):25. doi: 10.1186/s40900-016-0039-6
15. St Peter SD, Tsao K, Spilde TL, et al. Single daily dosing ceftriaxone and metronidazole vs standard triple antibiotic regimen for perforated appendicitis in children: a prospective randomized trial. *Journal of pediatric surgery* 2008;43(6):981-5. doi: 10.1016/j.jpedsurg.2008.02.018 [published Online First: 2008/06/19]
16. Guyatt GH, Oxman AD, Kunz R, et al. GRADE guidelines: 2. Framing the question and deciding on important outcomes. *J Clin Epidemiol* 2011;64(4):395-400. doi: 10.1016/j.jclinepi.2010.09.012 [published Online First: 2011/01/05]
17. Drake FT, Mottey NE, Farrokhi ET, et al. Time to appendectomy and risk of perforation in acute appendicitis. *JAMA Surg* 2014;149(8):837-44. doi: 1885706 [pii];10.1001/jamasurg.2014.77 [doi]
18. Boers M, Kirwan JR, Wells G, et al. Developing Core Outcome Measurement Sets for Clinical Trials: OMERACT Filter 2.0. *Journal of Clinical Epidemiology* 2014;67(7):745-53. doi: https://doi.org/10.1016/j.jclinepi.2013.11.013

AUTHORS' CONTRIBUTIONS

FS, SE and NH developed a first draft of the protocol. All authors advised on the development of the protocol and contributed towards the revision of the protocol for final submission.

FUNDING STATEMENT

The CONTRACT study is supported by the United Kingdom National Institute for Health Research Health Technology Assessment Programme (Grant number: 14/192/90 http://www.nets.nihr.ac.uk/projects/hta/1419290).

COMPETING INTERESTS STATEMENT

The authors report no competing interests.

ACKNOWLEDGEMENTS

All authors gratefully acknowledge funding from the National Institute for Health Research Health Technology Assessment Programme the CONTRACT trial, including the COS Development. SE gratefully acknowledges funding from Great Ormond Street Children's Charity.

Confidential: For Review Only

Confidential: For Review Only

APPENDIX 1. Systematic review of published outcomes of conservative treatment of acute appendicitis in children

All papers identified by our systematic review of conservative treatment[1] were included, and the literature search in that paper repeated to identify any further papers published between 2016 and April 2017.

Confidential: For Review Only

Table 1. Characteristics of included studies

Study	Publication Year	Region	Study Type	Sample size (intention to treat non-operatively)	Age of included population
Abes[2]	2007	Antibiotics	Retrospective	Middle East	16
Armstrong[3]	2014	Antibiotics vs. appendectomy	Retrospective	North America	12
Caruso[4]	2017	Antibiotics vs. appendectomy	Retrospective	Europe	197
Chen[5]	2016	Antibiotic	Retrospective	Asia	125
Gorter[6]	2015	Antibiotics	Prospective	Europe	25
Hartwich[7]	2016	Antibiotics	Prospective	North America	24
Jimbo[8]	2016	Antibiotics	Retrospective	Asia	71
Kaneko[9]	2004	Antibiotic	Prospective	Asia	91
Koike[10]	2014	Antibiotics	Retrospective	Asia	134
Mahida[11]	2016	Antibiotics	Prospective	North America	5
Minecci[12]	2016	Antibiotics	Prospective	North America	37
Mudri[13]	2017	Antibiotics and appendectomy	Retrospective	North America	26
Steiner[14]	2015	Antibiotics	Retrospective	Middle-east	45
Steiner[15]	2017	Antibiotics	Retrospective	Middle-east	197
Svensson[16]	2015	Antibiotics and appendectomy	Prospective RCT	Europe	24
Tanaka[17]	2015	Antibiotics and appendectomy	Retrospective	Japan	78

Outcomes from these papers were then mapped, where possible, to the outcomes identified in our previous systematic review of outcomes of appendicitis in children[18].

Confidential: For Review Only

Table 2. Outcomes mapped to those previously described[18]

	Abes	Armstrong	Caruso	Chen	Gorter	Hartwich	Jimbo	Kaneko	Koike	Mahida	Minecci	Mudri	Steiner 2015	Steiner 2017	Svensson	Tanaka
**Wound Infection**			x		x								x			
**Intra-abdominal abscess**			x	x	x							x				
**(Adhesive) obstruction**			x													
**Complication of antibiotics or treatment intervention**					x											
**Major/minor complications**		x														
**Non-infectious wound complications**																
**Re-admission to hospital**		x	x		x	X					x	x				
**Other infectious complications**		x		x								x				
**Interventional radiology procedure**																
**Need for operation/re-operation**			x		x	x	x	x		x	x	x	x	x	x	x
**Conversion laparoscopic to open surgery**																
**Recurrent appendicitis**	x	x			x	x	x	x	x	x	x	x	x	x	x	x
**Other complications**			X		x											
**Bacterial isolates**																
**Post-treatment fever**													x	x		
**Measure of recovery of gastro-intestinal function**							x									
**Blood markers (WCC/CRP)**			x										x	x		
**Duration of abdominal drainage**																
**Validated pain score**																
**Other pain assessment**	x		x										x		x	

Confidential: For Review Only

**Time away from normal activities or school**											x					
**Recovery to full activity/sport**																
**Cosmesis**																
**Time to ambulation**																
**Other PROM**																x
**Duration of home healthcare**																x
**Paediatric quality of life assessment**						x					x					
**Parental quality of life assessment**						x					x					
**Hospital length of stay**		x	x	x			x		x		x	x	x	x	x	x
**Duration of surgery/analgesia**							x									
**Total charges**						x			x		x	x			x	
**Duration of antibiotics**														x		
**Narcotic/analgesia doses**																
**Healthcare visits**		x														
**Post-treatment imaging**	x		x					x					x	x		x
**Cost effectiveness**						x	x					x				
**Death**																

No completely new outcomes were identified using this search. However, perforation is considered as a major complication, and it may be required to elevate this to the level of a separate outcome in the Core outcome set.

Confidential: For Review Only

References

1. Georgiou R, Eaton S, Stanton MP, et al. Efficacy and Safety of Nonoperative Treatment for Acute Appendicitis: A Meta-analysis. Pediatrics 2017;**139**(3).
2. Abes M, Petik B, Kazil S. Nonoperative treatment of acute appendicitis in children. J Pediatr Surg 2007;**42**(8):1439-42.
3. Armstrong J, Merritt N, Jones S, et al. Non-operative management of early, acute appendicitis in children: Is it safe and effective? Journal of Pediatric Surgery 2014;http://dx.doi.org/10.1016/j.jpedsurg.2014.02.071.
4. Caruso AM, Pane A, Garau R, et al. Acute appendicitis in children: not only surgical treatment. J Pediatr Surg 2017;**52**(3):444-48.
5. Chen CL, Chao HC, Kong MS, et al. Risk Factors for Prolonged Hospitalization in Pediatric Appendicitis Patients with Medical Treatment. Pediatrics and neonatology 2017;**58**(3):223-28.
6. Gorter RR, van der Lee JH, Cense HA, et al. Initial antibiotic treatment for acute simple appendicitis in children is safe: Short-term results from a multicenter, prospective cohort study. Surgery 2015;**157**(5):916-23.
7. Hartwich J, Luks FI, Watson-Smith D, et al. Nonoperative treatment of acute appendicitis in children: A feasibility study. J Pediatr Surg 2016;**51**(1):111-6.
8. Jimbo K, Takeda M, Miyata E, et al. Is a pediatrician performed gray scale ultrasonography with power Doppler study safe and effective for triaging acute non-perforated appendicitis for conservative management? J Pediatr Surg 2016;**51**(12):1952-56.
9. Kaneko K, Tsuda M. Ultrasound-based decision making in the treatment of acute appendicitis in children. J Pediatr Surg 2004;**39**(9):1316-20.
10. Koike Y, Uchida K, Matsushita K, et al. Intraluminal appendiceal fluid is a predictive factor for recurrent appendicitis after initial successful non-operative management of uncomplicated appendicitis in pediatric patients. Journal of Pediatric Surgery 2014;**49**(7):1116-21.
11. Mahida JB, Lodwick DL, Nacion KM, et al. High failure rate of nonoperative management of acute appendicitis with an appendicolith in children. J Pediatr Surg 2016;**51**(6):908-11.
12. Minneci PC, Mahida JB, Lodwick DL, et al. Effectiveness of Patient Choice in Nonoperative vs Surgical Management of Pediatric Uncomplicated Acute Appendicitis. JAMA Surg 2016;**151**(5):408-15.
13. Mudri M, Coriolano K, Butter A. Cost analysis of nonoperative management of acute appendicitis in children. J Pediatr Surg 2017.
14. Steiner Z, Buklan G, Stackievicz R, et al. A role for conservative antibiotic treatment in early appendicitis in children. J Pediatr Surg 2015;**50**(9):1566-8.
15. Steiner Z, Buklan G, Stackievicz R, et al. Conservative treatment in uncomplicated acute appendicitis: reassessment of practice safety. Eur J Pediatr 2017;**176**(4):521-27.
16. Svensson JF, Patkova B, Almstrom M, et al. Nonoperative treatment with antibiotics versus surgery for acute nonperforated appendicitis in children: a pilot randomized controlled trial. Ann Surg 2015;**261**(1):67-71.
17. Tanaka Y, Uchida H, Kawashima H, et al. Long-term outcomes of operative versus nonoperative treatment for uncomplicated appendicitis. J Pediatr Surg 2015;**50**(11):1893-7.
18. Hall NJ, Kapadia MZ, Eaton S, et al. Outcome reporting in randomised controlled trials and meta-analyses of appendicitis treatments in children: a systematic review. Trials 2015;**16**:275.

Confidential: For Review Only
